# Characterization and Antibacterial Properties of Polyetherketoneketone Coated with a Silver Nanoparticle-in-Epoxy Lining

**DOI:** 10.3390/polym14142906

**Published:** 2022-07-17

**Authors:** Wei-Fang Lee, Lu-Ying Wang, Ting-Yi Renn, Jen-Chang Yang, Lih-Sheng Fang, Yi-Huan Lee, Pei-Wen Peng

**Affiliations:** 1 School of Dental Technology, Taipei Medical University, Taipei 11031, Taiwan; weiwei@tmu.edu.tw; 2 Institute of Organic and Polymeric Materials, National Taipei University of Technology, Taipei 10608, Taiwan; ying3650@gmail.com; 3 Department of Oral and Maxillofacial Pathobiology, Graduate School of Biomedical and Health Sciences, Hiroshima University, Hiroshima 739-8511, Japan; tyrenn@hiroshima-u.ac.jp; 4 Graduate Institute of Nanomedicine and Medical Engineering, College of Biomedical Engineering, Taipei Medical University, Taipei 11052, Taiwan; yang820065@tmu.edu.tw; 5 Gijia Dental Lab, New Taipei City 23562, Taiwan; 600116@gmail.com

**Keywords:** polyetheretherketone, polyetherketoneketone, silver nanoparticles, *Porphyromonas gingivalis*

## Abstract

Polyetherketoneketone (PEKK) is an alternative material for use in removable partial denture frameworks; these frameworks must exhibit antibacterial properties to reduce the risk of periodontal disease. In the present study, silver nanoparticles (AgNPs) were synthesized via the reduction of silver nitrate with sodium borohydride in a solution containing polyvinyl pyrrolidone (PVP). Transmission electron microscope images and dynamic light scattering confirmed that metallic nanoparticles had been created with an average size of 32 nm. Furthermore, the coating of the PEKK polymeric substrate with 0.5% AgNPs was carried out using an epoxy resin lining at room temperature. Fourier transform infrared (FTIR) spectra confirmed the successful transfer of the AgNP-in-resin lining onto the polymeric substrate. Scanning electron microscopy and atomic force microscopy confirmed that the AgNPs had been uniformly deposited onto the PEKK specimens. Finally, the antibacterial activity of the specimens was tested against *Porphyromonas gingivalis*. An inhibition zone of 22.5 mm and an antibacterial rate of 83.47% were found for the PEKK coated with 0.5% AgNPs (0.5% Ag-PEKK) compared to an untreated polyetheretherketone (PEEK) substrate, evidencing that 0.5% Ag-PEKK has potential antibacterial properties for implant applications.

## 1. Introduction

Demands for biocompatible materials for dentition defects or loss, bone fractures, and joint replacements have boomed due to the global population’s increasing age longevity [1,2]. Metals and their alloys are primarily used as hard-tissue substitutes because of their successful clinical performance, which results from their excellent mechanical properties and corrosion resistance. However, the unaesthetic appearance, the mismatch of elastic moduli between biometallic materials and bone tissues, and the allergenic issues of these materials have encouraged researchers and scientists to seek new biomaterials to resolve such problems. Recently, advances in polymer-based materials have helped improve their biocompatibility and durability. Meanwhile, aesthetic appearances and flexible operations have expanded their applications as load-bearing implants [3].

Poly(aryl-ether-ketone) (PAEK) shows ultra-high performances in chemical resistance and tribological reliability among all thermoplastic composites [4]. Polyetheretherketone (PEEK) is one of the essential materials in this family. PEEK has an elastic modulus which is very close to that of cortical bone, with natural radiolucency also. Since being first introduced in the dental field in 1992, PEEK composite materials have been increasingly used as an alternative material for removable partial denture (RPD) frameworks with successful clinical outcomes [5,6]. PEEK was especially suitable for RPDs as clamps due to its low specific weight, nonmetallic taste, and high fatigue strength [7,8].

Polyetherketoneketone (PEKK), another member of the PAEK family, has been successfully applied for various industrial and military purposes since the 1960s [9]. PEKK and PEEK exhibit similar mechanical properties and similar whitish-gray appearances. The lack of methacrylate groups in the chemical structure improves the biocompatibility of PEKK. The presence of a second ketone group increases its structural variations [10]. After the promising applications of PEEK in dental implants, fixed prostheses, and removable dentures [11,12,13], PEKK was considered a potential biomaterial candidate. The potential applications of PEKK are similar to those of PEEK, and it has been approved by the U.S. Food and Drug Administration as an implantable material [14].

When used in implantable devices, PEKK should have appropriate mechanical properties and possess antibacterial properties to avoid biofilm colonization. However, PEKK does not exhibit antibacterial activity for preventing implant inflammation, which is one of the factors that result in revision surgeries [15]. Incorporating silver nanoparticles (AgNPs) onto the PEKK surface is an effective strategy to improve its antibacterial properties; however, solid adhesive bonding between AgNPs and PEKK is a challenging process due to its excellent chemical resistance [16]. Epoxy resin is one of the thermosetting resins which can easily interdiffuse into PEKK to form a semi-interpenetrating polymer network [17,18,19]. Adding AgNPs to epoxy resin improved the mechanical properties of epoxy composites and created physical crosslinking with PEEK, yet such studies are limited [20,21].

Under the above considerations, in the present study, AgNPs were chemically synthesized using a reduction–oxidation reaction and incorporated onto the PEKK surface using an epoxy lining. The AgNPs were characterized. The surface characterizations and in vitro antibacterial activity of PEKK coated with an AgNP-in-epoxy lining were compared to PEEK coated with an AgNP-in-epoxy lining.

## 2. Materials and Methods

### 2.1. Preparation and Characterization of AgNPs

Silver nitrate (AgNO_3_, 99.9%), sodium borohydride (NaBH_4_), and polyvinyl pyrrolidone (PVP, M_w_ = 40,000 mol.wt) were purchased from Sigma-Aldrich (St. Louis, MO, USA) and used without purification. Deionized water was used throughout our studies.

**Figure 1:**
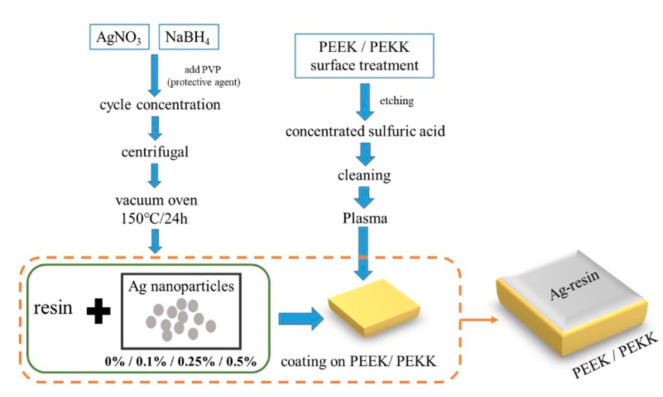
Schematic diagram of the process.

Figure 1 shows the schematic diagram of the process of the present study. AgNO_3_ (34 mg) was dissolved in 10 mL of distilled water with magnetic stirring to obtain a uniform solution. Ag^+^ ions were reduced to Ag^0^ atoms by chemical reduction using dropwise addition into 30 mL of 2 mM NaBH_4_ at 1 drop per second, which was placed in an ice bath, as shown in the following equation [22,23]:

$$2{\mathrm{AgNO}}_{3}+2{\mathrm{NaBH}}_{4}\to 2\mathrm{Ag}+2{\mathrm{NaNO}}_{3}+B_{2}H_{6}+H_{2}$$

After completion of the reaction, AgNPs were stored in a PVP aqueous solution, which served as the agent for stabilizing the AgNPs.

The formation of Ag^0^ atoms was monitored using an ultraviolet-visible (UV-vis) spectrophotometer (V-730, Jasco, Tokyo, Japan) with a 1 nm resolution from 300 to 800 nm. The average particle size and size distribution of AgNPs were measured in triplicate using dynamic light scattering (DLS, 90 Plus PALS, NanoBrook, Brookhaven, Holtsville, NY, USA). Transmission electron microscopic (TEM) images were obtained by placing a drop of an aqueous suspension of synthesized AgNPs onto a 300-mesh carbon-coated TEM copper grid and dried at ambient conditions at 200 kV (JEM2100F, JEOL, Tokyo, Japan). Chemical surface analyses of AgNPs were determined using electron spectroscopy for chemical analysis (ESCA) with an Al Kα X-ray source (JPS-9030, JEOL).

### 2.2. Characterization of Specimens Coated with an AgNP-in-Epoxy Lining

Two polymer blanks, PEEK (BreCAM, BioHPP, Bredent, Senden, Germany) and PEKK (Pekkton ivory, Cendres + Métaux SA, Biel/Bienne, Switzerland), were used as substrates. A precise low-speed cutter (Isomet, Buehler, Lake Bluff, IL, USA) was used to slice the PEEK and PEKK blanks into square specimens with dimensions of 10 mm × 10 mm × 2 mm. In total, five specimens for each material were used for the bacterial experiments. All specimens were ground with 1000-grit silicon carbide abrasive paper, and then were ultrasonically cleaned with acetone and deionized water for 5 min each and dried naturally. Afterwards, the specimens were further treated with argon plasma for 2 min.

As shown in Figure 1, AgNPs were collected from the PVP aqueous solution by distillation under reduced pressure and dried in the oven at 120 °C. Afterwards, the synthesized AgNPs of 0.25 and 0.5 mg were added to 100 mg of epoxy resin by stirring and sonication for 60 s and were sprayed onto PEEK or PEKK specimens, followed by heat-treatment in an oven at 120 °C for 20 min. For easy identification, PEKK coated with AgNPs of 0.25 mg and 0.5 mg were denoted as 0.25% Ag-PEKK and 0.5% Ag-PEKK.

The surface morphology and surface analysis of Ag-PEEK and Ag-PEKK were observed and measured using scanning electron microscopy (SEM), and the chemical compositions were detected by energy dispersive spectrometry (EDS, AZTecOne, Oxford Instruments, Abingdon, Oxfordshire, UK). The three-dimensional morphologies and roughness of the specimen surfaces were characterized using atomic force microscopy (AFM, XE-100, Park System, Santa Clara, CA, USA). The crystal phases of the nanoparticles and specimens with or without AgNP-in-epoxy lining coatings were measured using X-ray diffraction (XRD) in angular ranges (2θ) of between 20° and 80°. Chemical bonding information of Ag-PEEK and Ag-PEKK was characterized using Fourier-transform infrared (FTIR) spectroscopy (FT/IR-4600, Jasco, Tokyo, Japan) [14,24,25]. The FTIR spectra were recorded from 800 to 2000 cm^−1^ at a resolution of 0.7 cm^−1^.

### 2.3. Bacteriostatic Assays In Vitro

The bacteriostatic properties against Gram-negative *Porphyromonas gingivalis* (33277; ATCC, Manassas, VA, USA) of Ag-PEEK and Ag-PEKK were evaluated. *Porphyromonas gingivalis* was cultured in brain heart infusion (BHI; Oxoid, Basingstoke, UK) agar supplemented with 5 μg/mL hemin and 1 μg/mL vitamin K1 under anaerobic condition at 37 °C. The concentration of the bacterial suspension was adjusted to 10^8^ colony-forming units (CFU) mL^−1^. All specimens were wiped with 75% alcohol, rinsed with deionized water thrice, and subjected to UV irradiation for 2 h.

The disc diffusion method was used to screen the antimicrobial activity [26]. A bacterial suspension (100 μL) was adjusted to an optical density of 0.1 at 600 nm (0.5 McFarland standards) and spread uniformly on a BHI agar plate supplemented with 5% defibrinated sheep blood. Sterile specimens were placed on an agar plate and incubated at 37 °C for 48 h. Tests were conducted in triplicate. The inhibition zones formed around the specimens were observed and measured using optical microscopy (Olympus BX51, Tokyo, Japan) and ImageJ software (National Institutes of Health, Bethesda, MD, USA).

For the bacterial adhesion plate colony count, 2 mL of a bacterial suspension was cocultured with sterile specimens added under anaerobic conditions for 48 h. After that, specimens were vigorously washed with certain amounts of sterilized phosphate-buffered saline (PBS) to detach adherent bacteria. The obtained bacterial suspensions were diluted with normal saline by multiples of 10, and then 10 µL was dropped onto a BHI agar plate and cultured anaerobically for 48 h. The experiment was conducted in triplicate. The antibacterial ratio was calculated using the following formula:

$$\frac{M_{PEEK\;subsrtate}-N_{specimens}}{N_{PEEK\;substrate}}\times 100\%$$

where $N_{PEEK\;substrate}$ is the average number of the bacteria colonies on the PEEK substrate and $N_{specimens}$ is the average number of bacterial colonies on the PEKK substrate, Ag-PEKK, or Ag-PEEK at the different Ag concentrations.

## 3. Results

### 3.1. Characterization of the Synthesized AgNPs

**Figure 2:**
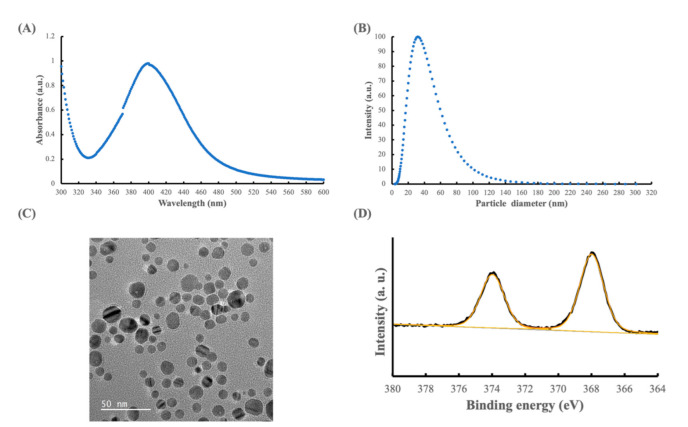
Characterization of sliver nanoparticles: (**A**) UV-Vis spectra, (**B**) particle size distribution graph characterized by dynamic light scattering, (**C**) transmission electron microscopic image, and (**D**) the deconvoluted X-ray photoelectron spectroscopic spectrum of the Ag 3d region.

The reduction of Ag^+^ to Ag^0^ by chemical reduction can be observed by a color change from transparent to yellowish-brown. The surface plasmon resonance of these Ag colloids with nano-sized particles displayed an intense absorption peak at a wavelength of 400 nm without additional peaks, as shown in Figure 2A. Studies showed that the surface plasmon resonance absorption band of AgNPs is located in the 350~450 nm region [27]. An increase in particle size and a shape change from spherical to irregular nanoparticles might shift the maximum absorption wavelength and cause the additional absorption peaks [28]. Figure 2A demonstrates the formation of AgNPs with no byproducts.

Figure 2B shows the results of the DLS measurements, revealing that the average hydrodynamic size of the synthesized AgNPs was 32 nm, and the average polydispersity value was 0.329, with an average zeta potential of −30.55 mV. The results indicated the synthesis of silver particles at a nano-size scale with moderate polydispersity. The zeta potential is an important factor in the stability of AgNPs in liquid suspensions, and the magnitude of the measured zeta potential indicated the presence of agglomerate particles [29]. In general, the AgNPs synthesized were negatively charged. The zeta potential for AgNPs was −30.55 mV, which we associated with the presence of PVP that provided steric resistance rather than electrostatic charge repulsion [30].

A TEM image of synthesized AgNPs is presented in Figure 2C and reveals that the distribution of the synthesized silver particles was uniform and well dispersed with little agglomeration. The TEM image further confirms that the synthesized AgNPs exhibited a spherical shape of 10~45 nm in diameter.

The wide scan range of the XPS image of the synthesized AgNPs demonstrated the presence of silver, oxygen, and carbon species, as shown in Figure 2D. The high-resolution spectrum of Ag 3d illustrated two asymmetrical peaks with well-separated spin-orbit components. The deconvoluted Ag 3d spectrum represented the binding energies of Ag 3d5/2 and Ag 3d3/2 at 368 and 373 eV, respectively, indicating that the synthesized AgNPs were primarily present in a metallic state [31].

### 3.2. Characterization of Specimens Coated with the AgNP-in-Epoxy Lining

**Figure 3:**
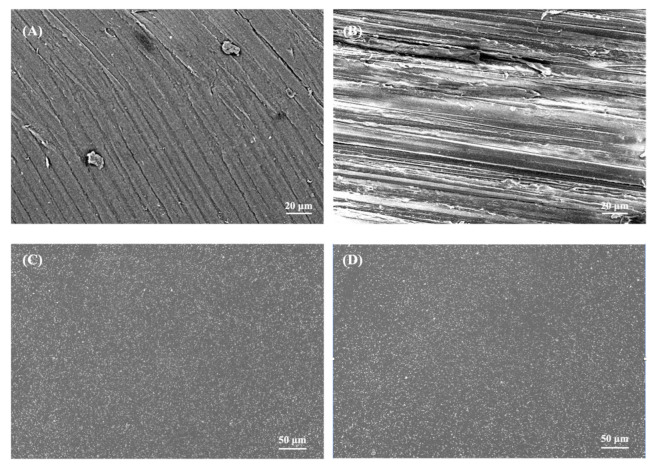
Scanning electron microscopy images of (**A**) polyetherketoneketone (PEKK), (**B**) polyetheretherketone (PEEK), (**C**) 0.5% silver nanoparticle (Ag)-PEKK, and (**D**) 0.5% Ag-PEEK.

Compared to uncoated substrates, the specimens coated with an AgNP-in-epoxy lining turned slightly gray due to the presence of AgNPs on the surface, which was similar to the findings of a previous study [1]. Figure 3 shows the SEM topography of the specimens. The surface of the uncoated PEKK and PEEK specimens exhibited regular patternings due to grinding and were rough, as shown in Figure 3A,B. After being coated with the AgNP-in-epoxy lining, relatively smooth surfaces with a homogeneous dispersion of particles were visible for both the PEKK and PEEK specimens, as shown in Figure 3C,D. The 0.25% Ag-PEKK and 0.5% Ag-PEEKK revealed similar surface morphologies.

**Figure 4:**
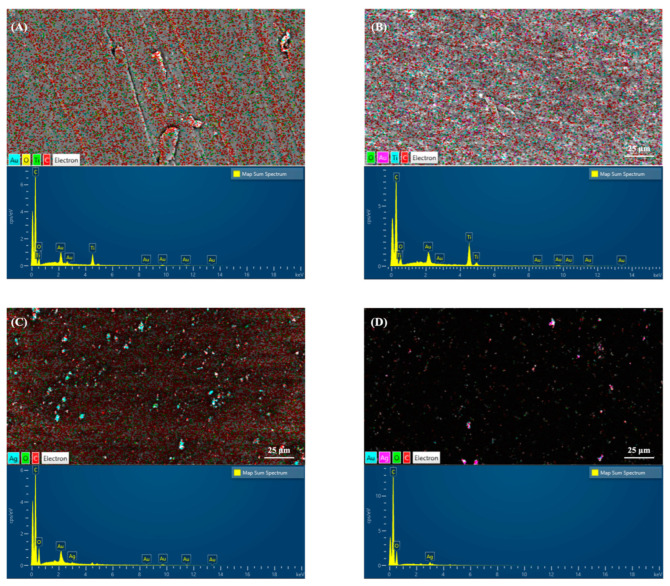
Energy-dispersive X-ray spectroscopic microanalysis of (**A**) polyetherketoneketone (PEKK), (**B**) polyetheretherketone (PEEK), (**C**) 0.5% silver nanoparticle (Ag)-PEKK, and (**D**) 0.5% Ag-PEEK.

Figure 4A,B show the EDS observations, confirming that 0.5% Ag-PEKK and 0.5% Ag-PEEK possessed sliver elements, unlike the untreated PEKK and PEEK surfaces, as shown in Figure 4C,D. The above observations showed that the AgNPs were uniformly arranged on both the PEKK and PEEK substrates.

**Figure 5:**
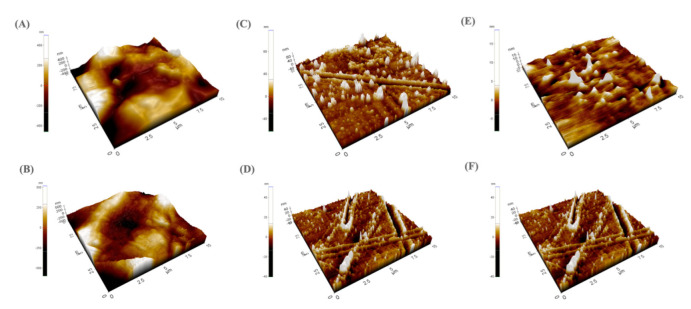
Atomic force microscopic images of (**A**) polyetherketoneketone (PEKK), (**B**) polyetheretherketone (PEEK), (**C**) 0.25% Ag−PEKK, (**D**) 0.25% Ag−PEEK, (**E**) 0.5% Ag−PEKK, and (**F**) 0.5% Ag−PEKK.

Figure 5 depicts the three-dimensional morphology of all specimens using AFM. The surfaces of the Ag-PEEK and Ag-PEKK specimens exhibited different morphologies from the uncoated substrates, confirming the SEM observations described above. The AgNPs were homogeneously embedded in the epoxy lining and coated onto the PEEK and PEKK substrates. The roughness of the specimens was evaluated via average roughness (Ra) and root mean square (RMS) parameters, as shown in Table 1. The roughness of the Ag-PEEK and Ag-PEKK specimens decreased when compared to the uncoated substrates. Furthermore, the specimen surface roughness decreased with the increased concentration of AgNPs. This finding could be explained by the fact that specimens covered with the AgNP-in-epoxy lining had a thickness of 3.57 mm, and the AgNPs were embedded in this lining. Liu et al. [1] prepared nano-silver-coated PEEK using magnetron sputtering. The excited silver ions produced a greater surface roughness under electric field and magnetic field acceleration. In the present study, the roughened surface of the uncoated PEKK and PEEK substrates resulted from argon plasma. In contrast, the epoxy, a reinforcing lining between AgNPs and the substrates, resulted in smoother surfaces [19]. The surface roughness of biomaterials highly affects bacterial responses, including their adhesion and spread. Generally speaking, smoother surfaces on a material better prevent bacterial adhesion and growth over its surface [32].

**Figure 6:**
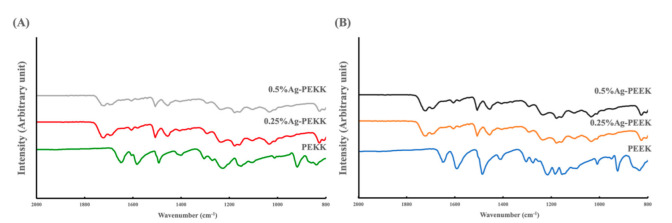
Fourier transform infrared (FTIR) of (**A**) polyetherketoneketone (PEKK) and (**B**) polyetheretherketone (PEEK) with different concentration of silver nanoparticles.

To verify the chemical composition of the AgNP-in-epoxy lining coated onto the PEEK and PEKK substrates, FTIR analyses were performed on specimens, and the results are shown in Figure 6 and Table 2, including results from previously published literature [14,24,33]. The PEKK and PEEK spectra showed similar characteristic bands, including diphenyl ketone bands, asymmetric stretching vibrations of C-O-C bonds of diaryl groups, and C=C stretching vibrations in benzene ring bands. Additional peaks from PEEK were noted, which corresponded to phenyl rings and a diphenyl ether group. These observations were similar to those of a previous study [10]. After being coated with the AgNP-in-epoxy lining, the spectra of Ag-PEKK and Ag-PEEK differed from those of uncoated substrates and exhibited the characteristic signals of Ag-in-epoxy lining. The characteristic signals of the PEKK or PEEK substrates were lost. Bands characteristic of the substrate were lost, with peaks corresponding to C-O-C stretching vibrations in ether groups and aromatic carbon and carbonyl stretching modes observed. These observations demonstrated that the AgNP-in-epoxy lining was successfully coated onto the polymeric substrates. The spectra of Ag-PEKK and Ag-PEEK displayed a similar pattern to that of the Ag-in-epoxy lining. This finding is evidenced by the fact that the specimens covered with the AgNP-in-epoxy lining had a thickness of 3.57 mm, with the AgNPs embedded in this thinning, while the penetration depth of FTIR is only a few micrometers.

**Figure 7:**
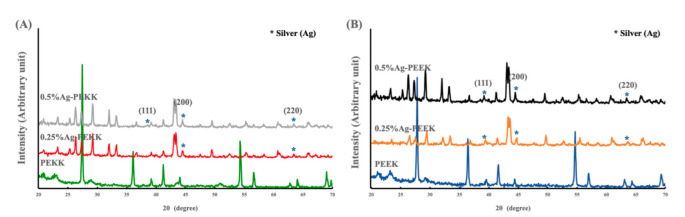
Diffractograms of (**A**) PEKK and (**B**) PEEK coated with the AgNP-in-epoxy lining, with different initial concentrations of AgNPs.

Figure 7 depicts the XRD patterns of PEKK and PEEK, which show the same characteristic peaks. The XRD patterns of Ag-PEKK and Ag-PEEK revealed characteristic peaks at 38.14°, 44.36°, and 64.42°, which were consistent with the Joint Committee on Powder standard powder diffraction card 04-0783 [29]. These characteristic peaks indicated that AgNPs of a high purity and stable size were present on the surface. With the increasing concertation of AgNPs, the intensity of these peaks increased.

Cruz-Pacheco et al. [21] immersed PEEK in an AgNPs-containing solution and used FTIR and TEM to demonstrate the presence of electrostatic interactions between the polymer and the AgNPs. The epoxy resin served as the lining in this study, easily interdiffusing into PEKK to form a semi-interpenetrating polymer network [17,18,19].

### 3.3. Antibacterial Properties of Specimens Coated with the AgNP-in-Epoxy Lining

**Figure 8:**
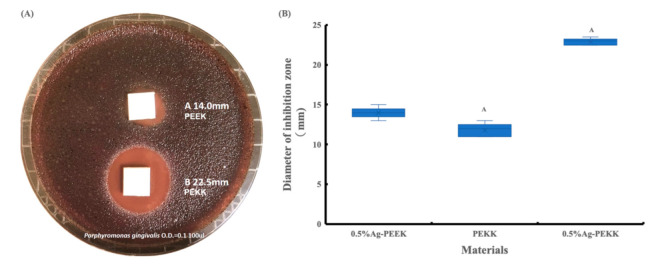
(**A**) Inhibition zones of the 0.5% silver nanoparticle (Ag)-polyetherketoneketone (PEKK) and 0.5% Ag-polyetheretherketone (PEEK), and (**B**) the diameter of the inhibition zone.

The disc diffusion method was conducted to preliminary screen for antibiotic activity of Ag-PEKK and Ag-PEEK against *P. gingivalis*, as shown in Figure 8. There was no inhibition zone around the PEKK substrate, whereas this was observed around the PEKK, Ag-PEKK, and Ag-PEEK surfaces. Figure 8 also shows the average diameters of the inhibition zones measured with ImageJ software. The 0.5% Ag-PEKK sample had significantly larger zones of inhibition among all groups (*p* < 0.05). This suggests that 0.5% Ag-PEKK and 0.5% Ag-PEEK possessed diffusion properties, which inhibited *P. gingivalis* growth. The PEEK substrate was considered as the control group for calculating the antibacterial rates.

Table 3 lists the antibacterial rates of the specimens. According to CFU counts, numerous bacteria adhered to the PEEK substrate, and more than 40% less *P. gingivalis* was found on the PEKK substrate. The PEKK and PEEK specimens exhibited antibacterial properties due to the presence of the AgNPs. The antibacterial properties increased with the increasing concentrations of AgNPs. The amounts of *P. gingivalis* were reduced by approximately 74% and 83% for 0.5% Ag-PEEK and 0.5% Ag-PEKK, respectively.

In the present study, *P. gingivalis* was used to evaluate the antibacterial activity of 0.5% Ag-PEKK and 0.5% Ag-PEEK. *Porphyromonas gingivalis* is a member of the red complex of periodontal bacteria. If oral and denture hygiene is not carefully monitored, the lingual cervical coverage of RPDs for the colonization of bacteria might increase the risk of periodontal disease [34].

According to in vitro antibacterial assessments, the PEEK substrate did not exhibit antimicrobial activity, which is consistent with a previous study [19]. Impressively, *P. gingivalis* grew to a lesser extent on the PEKK substrate compared to the PEEK substrate. Wang et al. [15] prepared 3D-printed PEKK with nanostructured surface features, which decreased the adhesion and growth of *P. aeruginosa* and *Staphylococcus epidermidis* compared to conventional PEEK surfaces.

In the PAEK family, PEKK and PEEK are critical materials. Both of them outperformed conventional biopolymers in terms of strength and rigidity. For the double-crown system, the PEKK/PEKK combination outperformed the cobalt-chrome/PEKK combination in a study by Igarashi et al. [12]. In another study, despite having lower retentive forces than CoCr clasps, the PEKK clasp maintained similar or higher retentive forces after 15,000 insertion and removal cycles [13]. As a result, PEKK could be used for RPDs, fixed partial dentures, or overdentures. Because both PEKK and PEEK specimens were industrially manufactured, a reasonable explanation for this could be the difference in their chemical characteristics. It was speculated that the additional ketone bonds in the PEKK chemical structure may decrease bacterial attachment and growth because of their versatile chemistry and altered surface energetics. Future studies need to investigate the influence of PEKK on the resultant antibacterial responses of different bacterial strains.

The antibacterial mechanism of AgNPs has been widely studied [35,36,37]. According to the deconvoluted Ag 3d spectrum results, the synthesized AgNPs were primarily present in a metallic state (Figure 2D). Ag^+^ ions, which are converted from AgNPs in the physiological environment, deform cell membranes and interact with some proteins [1]. Rtimi et al. [37] proposed a new perspective. They concluded that the interaction between Ag-sputtered materials and bacteria, through direct surface contact-induced redox reaction, resulted in an increase in the permeability of the bacterial cell envelope and the modification of the interfacial bacterial potential. Furthermore, Ag+ ions accelerated bacterial inactivation. Therefore, the mechanism for antibacterial properties on Ag-PEKK specimens still needs to be explored under different incubation periods in future studies. Future studies also need to investigate the influence of PEKK coated with the AgNP-in-epoxy lining against different bacterial species. In addition, an investigation of the cytotoxic effect of PEKK coated with the AgNP-in-epoxy lining at different concentrations is required in future studies.

## 4. Conclusions

A simple method for the inhibition of bacterial growth using PEKK and PEEK coated with AgNPs was explored for promising implantation applications. Well-dispersed silver particles with a spherical shape and an average hydrodynamic size of 32 nm were prepared by reducing silver nitrate with sodium borohydride in the presence of the protective agent PVP. Results of the in vitro study indicated potential antibacterial ability against *P. gingivalis* on 0.5% Ag-PEKK and 0.5% Ag-PEEK surfaces. The results of the present study show that one can reduce bacterial functions via a simple coating of an AgNP-in-epoxy lining on PEKK, which should be further studied for a wide range of antibacterial biomedical applications.

## Figures and Tables

**Table 1:** Surface roughness values of all specimens.

	Surface Roughness	
	Ra (nm)	RMS (nm)
PEKK	120.262	148.744
0.25% Ag-PEKK	2.914	4.825
0.5% Ag-PEKK	1.391	2.002
PEEK	115.988	149.957
0.25% Ag-PEEK	4.939	6.950
0.5% Ag-PEEK	2.555	4.743

Ra: average roughness; RMS: root mean square; PEKK: polyetherketoneketone; PEEK: polyetheretherketone.

**Table 2:** Characteristic absorption peaks observed in FTIR spectra of all specimens.

Peak Location (cm^−1^)	Bond Type	Specimen
918	Diphenyl ketone group	PEKK, PEEK
1033	C-O-C stretching vibrations in ether groups	Ag-PEKK and Ag-PEEK
1150	Asymmetric stretching vibration of C−O−C bonds in diaryl groups	PEKK, PEEK
1182	Diphenylether group	PEEK
1215	Diphenylether group	PEEK
1220	Asymmetric stretching vibrations of C−O−C bonds in diaryl groups	PEKK, PEEK
1232	C-O-C stretching vibrations in ether groups	Ag-PEKK and Ag-PEEK
1485	Phenyl rings of diphenylether group	PEEK
1492	Diphenyl ketone group	PEKK, PEEK
1507	Aromatic carbon	Ag-PEKK and Ag-PEEK
1583	C = C stretching vibrations in benzene ring bands	PEKK, PEEK
1594	Phenyl rings	PEEK
1647	Diphenyl ketone group	PEKK, PEEK
1722	Carbonyl stretching mode	Ag-PEKK and Ag-PEEK

PEKK: polyetherketoneketone; PEEK: polyetheretherketone; Ag: silver nanoparticle.

**Table 3:** Antibacterial activities of the specimens.

	PEEK	0.25% Ag-PEEK	0.5% Ag-PEEK	PEKK	0.25% Ag-PEKK	0.5% Ag-PEKK
Porphyromonas gingivalis (CFU)	1411.6 ± 68.2	538.6 ± 30.2	358.3 ± 38.2	641.7 ± 34.0	500.3 ± 29.6	233.3 ± 30.6
Antibacterial rate (%)	-	61.87	74.63	54.57	64.56	83.47

PEEK: polyetheretherketone; Ag: silver nanoparticle; PEKK: polyetherketoneketone.

## Data Availability

Not applicable.
